# Psychological impact and quality of life in pediatric patients with chronic skin disorders: a systematic review (2010–2025)

**DOI:** 10.3389/fped.2026.1821918

**Published:** 2026-07-03

**Authors:** Abdulaziz Fahd AlKaabba, Shahd Muhammed Aldosari, Taif Abdullah Alwadai, Aldana Abdulrahaman Alodayani, Duna Saad Alhumaidan, Wajed Shuliweeh Alenezi, Renad Sami Almuharib, Renad Abdullah Almosa

**Affiliations:** Department of Family and Community Medicine, College of Medicine, Imam Mohammad Ibn Saud Islamic University (IMSIU), Riyadh, Saudi Arabia

**Keywords:** caregiver burden, chronic skin disorders, health-related quality of life, pediatric dermatology, psychological distress

## Abstract

**Introduction:**

Chronic skin disorders in children and adolescents are increasingly recognized as conditions with significant psychosocial implications. Beyond physical manifestations, these diseases adversely affect psychological well-being, health-related quality of life (HRQoL), and family functioning.

**Methods:**

A systematic review was conducted following PRISMA guidelines. Five electronic databases were searched for studies published between 2010 and 2025. Empirical studies assessing psychological outcomes or HRQoL among pediatric patients (≤18 years) with chronic dermatologic conditions were included. Methodological quality was appraised using JBI and CASP tools. Due to heterogeneity in study designs and outcome measures, findings were synthesized narratively.

**Results:**

A total of 1,844 records were identified, with 41 studies meeting inclusion criteria. Most studies were cross-sectional (approximately 75%), with 12 classified as high methodological quality. Sample sizes ranged from fewer than 10 to over 11,000 children. Across conditions including atopic dermatitis, psoriasis, vitiligo, alopecia areata, hidradenitis suppurativa, and congenital ichthyosis, elevated rates of anxiety, depression, stigma, and emotional distress were consistently reported. Severe atopic dermatitis was associated with nearly a two-fold increased risk of depressive and internalizing symptoms in longitudinal data. HRQoL impairment commonly affected emotional functioning, peer relationships, school participation, and sleep. Caregiver burden was documented in 19 studies.

**Discussion:**

Findings highlight the multidimensional burden of pediatric chronic skin disease, where psychosocial distress often parallels physical symptom severity.

**Conclusion:**

Chronic skin disorders are consistently associated with psychological distress and reduced HRQoL. Causal inferences cannot be drawn given cross-sectional designs; findings support routine psychosocial assessment and family-centered care to improve outcomes.

## 1 Introduction

Chronic skin diseases in childhood and adolescence are usually associated with considerable physical discomfort and functional impairment, but emerging evidence indicates that their implications reach much further, impacting psychological well-being, emotional adjustment, social acceptance, and quality of life almost since the outset of life. Conditions such as atopic dermatitis, psoriasis, vitiligo, alopecia areata, congenital ichthyosis and chronic urticaria often begin early in life and take the course of a relapsing or persistent disease exposing children and adolescents to long-term psychosocial stress during critical times in their development (1, 2). Pediatric dermatology is thus increasingly becoming an interdisciplinary specialty that rests at the interface of psychiatry and psychology, behavioral sciences and family medicine.

Chronic inflammatory and autoimmune skin diseases pose a significant public health burden globally in children, with atopic dermatitis alone affecting up to 20% of the world's children, and psoriasis, vitiligo and alopecia areata affecting millions more (3, 4). These disorders are frequently associated with pruritus, pain, sleep disturbance, and recurrent infections, which interfere with daily activities, school attendance, and peer relationships. More importantly, visible skin lesions may provoke social stigma, bullying, and discrimination, leading to social withdrawal and reduced self-esteem (5, 6). Such experiences place affected children at increased risk of anxiety, depressive symptoms, emotional dysregulation, and impaired psychosocial functioning.

Recent studies have underscored strong bidirectional relationships between dermatological disease activity and psychological wellbeing. Emotional distress exacerbates inflammatory responses and amplifies disease severity, whilst uncontrolled symptoms add to the emotional burden (7, 8). This vicious cycle occurs often in chronic relapsing conditions such as atopic dermatitis and psoriasis, exacerbating disease flares and poor treatment adherence, due to stress and sleep disruption and maladaptive coping strategies. Time-varying vulnerability to adverse effects on psychological wellbeing is often present in pediatric patients, and this vulnerability may persist into adulthood, if unrecognized.

Health-related quality of life (HRQoL) has increasingly been recognized as a central outcome of interest in pediatric dermatology. Beyond purely clinical metrics, HRQoL estimates improve understanding of the stir-causal interconnectedness of multidimensional impacts of disease upon physical comfort, emotional wellbeing, social participation, school functioning, family dynamics, among others. Several instruments including the Children's Dermatology Life Quality Index (CDLQI), Pediatric Quality of Life Inventory (PedsQL), etc., as well as Skindex, have come into broad use to assess these domains (9, 10). Empirical studies conducted across diverse cultural contexts have consistently reported moderate to severe impairment in HRQoL among children with chronic skin disorders, particularly in emotional and social domains (11, 12).

Importantly, the psychological impact of pediatric skin disease has not been confined to patients alone. Parents and caregivers frequently experience elevated levels of stress, anxiety, depression, and caregiver burden as a result of long-term disease management, financial strain, and concerns about stigma and prognosis (13, 14). Family functioning, parental coping styles, and caregiver mental health collectively influence children's treatment adherence and psychological adjustment. Accordingly, chronic skin disorders have recently been viewed as family-centered disorders that can be best managed by a holistic approach.

Despite the expanding body of literature in this field, several limitations have persisted. First, many studies have focused on single disease entities or specific outcomes, limiting the generalizability of findings across dermatological conditions. Second, substantial heterogeneity exists in study designs, assessment tools, and outcome definitions, which complicates cross-study comparisons (15). Third, a considerable proportion of published research has emphasized biomedical outcomes, with relatively limited integration of psychosocial, behavioral, and contextual factors. Fourth, previous reviews have often combined pediatric and adult populations, obscuring age-specific vulnerabilities and developmental considerations.

Moreover, existing systematic reviews published before 2010 have not consistently incorporated recent advances in psychodermatology, digital health monitoring, patient-reported outcome measures, and family-centered interventions. Several reviews have focused primarily on prevalence estimates or instrument validation, without comprehensive synthesis of risk and protective factors, coping mechanisms, and clinical implications (7, 16). As a result, clinicians and policymakers have lacked consolidated, updated evidence to guide integrated psychosocial care in pediatric dermatology.

Within this context, there has been a growing call for systematic, methodologically rigorous syntheses that examine psychological outcomes and quality of life across diverse pediatric skin disorders. Recent clinical guidelines and expert consensus statements have emphasized the need for routine mental health screening, early psychosocial intervention, and multidisciplinary collaboration (17, 18). However, implementation remains inconsistent, partly due to fragmented evidence and limited clarity regarding high-risk subgroups and modifiable protective factors.

Based on the protocol and proposal guiding the present review, several key gaps were identified. These included: (1) insufficient comparative analysis of psychological outcomes across different chronic skin conditions; (2) limited synthesis of risk and protective factors influencing psychological resilience; (3) inconsistent reporting of HRQoL domains and measurement tools; and (4) inadequate translation of research findings into practical clinical recommendations. Addressing these gaps was essential to advance holistic pediatric dermatology care and inform evidence-based policy development.

As a result, the systematic literature review examined the body of existing empirical literature relating to the psychological impact and health-related quality of life (HRQoL) of children with chronic skin conditions between 2010 and 2025. The primary purpose of the review was to assess the nature and extent of the psychological impact encountered by children and adolescents suffering from chronic dermatological conditions. The review specifically aimed to meet the following objectives: (1) to classify and identify psychological outcomes among various types of diseases; (2) to evaluate the impact chronic skin disorders have on HRQoL across all domains; (3) to investigate both risk and protective factors that affect children's psychological well-being; and (4) to create clinical, mental health, and caregiver education recommendations based upon the evidence collated within this systematic review.

The intention of this systematic review was to synthesize all of the evidence from quantitative, qualitative, and mixed methods research proposals to date. As opposed to previous systematic reviews, the primary focus of this review was on pediatric patients, and a rigorous inclusion/exclusion criterion was applied in order to ensure that the evidence included contained equivalent measures, as well as reflecting the psychosocial dimension of the participant subjects, and not solely the clinical measures and/or reporting.

As a result, the evidence will provide the foundation for the development of multi-disciplinary, patient-focused, holistic models of care delivery which provide for the comprehensive treatment of patients' physical symptoms and psychological needs. In summary, this systematic review will provide a unique contribution to the body of scientific knowledge relating to the complex interplay of chronic skin disease with childhood and adolescent mental health and HRQoL.

## 2 Methods

### 2.1 Study design and protocol

This study was performed as a systematic review of the psychological aspects and health-related quality of life of children and adolescents with dermatological conditions (health issues) between the ages of 0–17 inclusive. The review was designed and conducted per Preferred Reporting Items for Systematic Reviews and Meta-Analyses (PRISMA 2020) guidelines. A detailed protocol was developed *a priori* based on the research proposal submitted to the research ethics committee and study aims. The protocol outlined the research questions, eligibility criteria, search strategy, outcome measures, and methods for quality appraisal and data synthesis. This helped to ensure methodological transparency and reproducibility. This review was initially planned for prospective registration in PROSPERO (National Institute for Health Research, 2022) as outlined in the study proposal.

However, due to administrative and timeline issues, formal registration of the review protocol was not completed prior to screening. All eligibility criteria, methodological procedures, and analytic decisions were nevertheless determined *a priori* in the approved protocol prior to study selection and data extraction, limiting the risk of selective reporting. There were no major deviations from the original protocol in the conduct of this review.

### 2.2 Information sources and search strategy

A systematic and extensive literature search was performed to identify studies published between 2010 and 2025. As per the approved protocol, the following electronic databases were searched: PubMed/MEDLINE, Scopus, Web of Science, PsycINFO and the Cochrane Library. Embase was also searched during the review process to enhance database coverage and ensure retrieval of relevant international dermatology and psychosocial literature.

The following search string was applied across all databases, with minor syntax adaptations to suit each platform:

(“chronic skin disease” OR “chronic dermatological condition” OR “atopic dermatitis” OR “eczema” OR “psoriasis” OR “vitiligo” OR “alopecia areata” OR “ichthyosis” OR “hidradenitis suppurativa” OR “chronic urticaria”) AND (“child” OR “children” OR “adolescent” OR “pediatric” OR “paediatric”) AND (“psychological impact” OR “psychological distress” OR “mental health” OR “anxiety” OR “depression” OR “emotional wellbeing” OR “quality of life” OR “health-related quality of life” OR “HRQoL” OR “caregiver burden” OR “stigma” OR “social functioning”)

Filters applied included: publication date 2010–2025, English language, and human subjects. No restrictions were placed on study design at the search stage, with design-based eligibility applied during screening.

Grey literature sources were initially screened in accordance with the protocol; however, no eligible empirical studies meeting the predefined inclusion criteria were identified from grey literature searches. Therefore, all included studies were derived from peer-reviewed journal publications.

Reference lists of eligible articles and relevant review papers were manually screened to identify further potentially eligible studies.

### 2.3 Eligibility criteria

Studies were selected based on predefined inclusion and exclusion criteria.

#### 2.3.1 Inclusion criteria

Studies were included if they met all of the following conditions:

- Involved children and adolescents aged 0–18 years with chronic dermatological conditions.
- Assessed psychological outcomes and/or health-related quality of life.
- Employed quantitative, qualitative, or mixed-methods designs.
- Were published in peer-reviewed journals between 2010 and 2025.
- Were available in full-text format in English.

While the original protocol specified psychological and HRQoL outcomes as eligibility criteria, priority was given during study selection to research utilizing validated and widely recognized measurement instruments (e.g., CDLQI, PedsQL, PHQ-9, GAD-7) in order to enhance methodological rigor and comparability across studies.

#### 2.3.2 Exclusion criteria

Studies were excluded if they:

- Focused exclusively on adult populations.
- Did not report psychological or quality-of-life outcomes.
- Were case reports, editorials, commentaries, conference abstracts, or letters.
- Lacked sufficient methodological detail or outcome data.
- Were duplicate publications.
- Were not accessible in full text.

### 2.4 Study selection process

All identified records were imported into a reference management system, and duplicates were removed. Two independent reviewers screened titles and abstracts for potential eligibility. Full-text articles were retrieved for studies that appeared relevant or where eligibility was uncertain.

Full-text screening was conducted independently by the same reviewers using the predefined inclusion and exclusion criteria. Discrepancies were resolved through discussion and, when necessary, consultation with a third reviewer. The study selection process was documented using a PRISMA flow diagram, detailing the number of records identified, screened, excluded, and included.

Following this process, a total of 41 studies met the eligibility criteria and were included in the final synthesis.

### 2.5 Data extraction

Data were extracted using a standardized and piloted data extraction form developed based on the study objectives and protocol. The following information was systematically collected from each included study:

- Author(s), year of publication, journal, and country
- Study design and setting
- Sample size and participant characteristics
- Type and severity of dermatological condition
- Psychological outcomes assessed
- Health-related quality-of-life domains
- Measurement instruments used
- Risk and protective factors
- Key findings
- Funding sources

Data extraction was performed independently by two reviewers. Extracted data were cross-checked for accuracy and completeness. Any inconsistencies were resolved by consensus.

### 2.6 Quality assessment and risk of bias

The methodological quality of included studies was evaluated using appropriate appraisal tools according to study design. Cross-sectional and observational studies were assessed using the Joanna Briggs Institute (JBI) Critical Appraisal Checklists. Qualitative studies were appraised with the Critical Appraisal Skills Programme (CASP) qualitative checklist while systematics reviews were assessed with the PRISMA-guided criteria.

Each study was rated as having low, moderate, or high risk of bias based on sampling methods, measurement validity, data analysis, and reporting transparency. Quality assessment was conducted independently by two reviewers, and disagreements were resolved through discussion.

### 2.7 Assessment of publication bias

As this review did not perform quantitative pooling due to substantial methodological and clinical heterogeneity across included studies, formal statistical assessment of publication bias (e.g., funnel plots or Egger's regression test) was not applicable. In order to mitigate publication bias, we adapted an extensive search strategy employed in our previous systematic reviews, which extended across six electronic databases (PubMed/MEDLINE, Scopus, Web of Science, Embase, PsycINFO, and the Cochrane Library) and were screened via reference lists of eligible studies and pertinent reviews to further identify non-indexed studies.

Study characteristics, declarations of funding sources, geographic distribution, and consistency of reported outcomes were checked for evidence of a selective reporting pattern. Including studies from multiple countries and multiple healthcare settings also lowers the likelihood of reporting bias.

However, despite these strengths, there are still limitations. We included only peer-reviewed full-text articles published in English and did not systematically search for conference abstracts, dissertations and other unpublished studies, which poses the risk for language and selective publication bias. We also did not prospectively register our review protocol in PROSPERO, and thus cannot exclude the possibility of selective reporting of outcomes across the included primary studies. Nonetheless, the overall consistency of findings across study designs and settings provides further confidence in these synthesized conclusions.

### 2.8 Outcome measures

The main outcomes of the review were psychological and psychosocial indicators, that is depression, anxiety, stress, self-esteem, stigma, social functioning and behavior difficulties. Secondary outcomes were health-related quality of life; across domains of physical, emotional, social, school and family.

Validated instruments commonly used across studies included the Children's Dermatology Life Quality Index (CDLQI), Infant's Dermatology Life Quality Index (IDQoL), Family Dermatology Life Quality Index (FDLQI), Pediatric Quality of Life Inventory (PedsQL), Patient Health Questionnaire (PHQ-9), Generalized Anxiety Disorder Scale (GAD-7), Children's Depression Inventory (CDI), and Strengths and Difficulties Questionnaire (SDQ).

### 2.9 Data synthesis and analysis

Given the heterogeneity of study designs, populations, dermatological conditions, and outcome measures, a meta-analysis was not conducted. Instead, a narrative synthesis approach was employed.

Findings were synthesized according to the following themes:

1. Type of dermatological condition
2. Psychological outcomes
3. Quality-of-life domains
4. Family and caregiver impact
5. Risk and protective factors
6. Outcomes in regard to interventions.

Quantitative results were narratively synthesized and reported using descriptive statistics where appropriate, such as prevalence rates, mean scores, and correlation coefficients. Qualitative findings were synthesized using thematic synthesis to describe common psychosocial patterns and experiences.

### 2.10 Ethical considerations

This systematic review was based solely on previously published data and did not collect data from human participants directly. Therefore, formal ethical approval for the review was not required. All studies included had obtained ethical clearance from their review boards, as reported in their original publications.

## 3 Result

### 3.1 Study selection

A comprehensive database search identified 1,844 records published between 2010 and 2025. After removal of duplicates (*n* = 328), records excluded by automation tools (*n* = 194), and records removed for other reasons (*n* = 124), 1,198 records were screened at the title and abstract level. Of these, 667 records were excluded.

A total of 531 reports were sought for retrieval, of which 285 were not retrieved, leaving 246 full-text articles assessed for eligibility. Following full-text review, 205 reports were excluded due to inadequate data, resulting in 41 studies included in the final synthesis.

**Figure 1:**
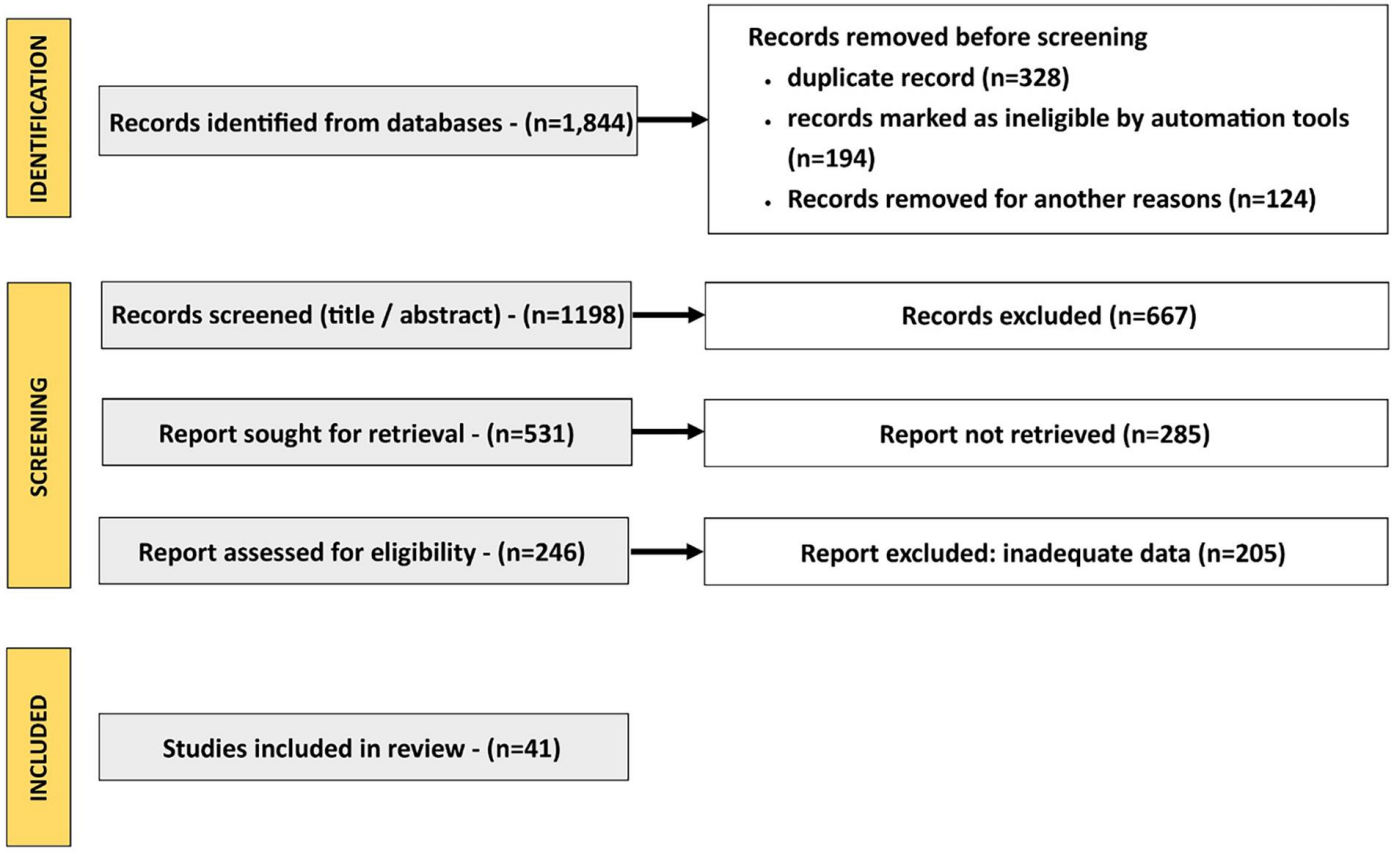
PRISMA flow diagram of study selection (2010–2025). PRISMA flow diagram illustrating the identification, screening, eligibility, and final inclusion of studies in the systematic review (2010–2025).

The study selection process followed PRISMA 2020 guidelines and is illustrated in Figure 1.

### 3.2 Study characteristics

The 41 identified studies were published between 2010 and 2025 and were primarily from Europe and Asia, but there also studies included from the Middle East, North America, and Africa (see Table 1). A majority of studies used cross-sectional designs, with few using longitudinal cohort, qualitative, case-control, psychometric validation, or experimental designs.

**Table 1:** Summary of included studies (*n* = 41).

No.	Author(s)	Year	Country/setting	Study design	Sample size	Age range	Condition	Main outcomes	QoL instrument
01	Aldosari et al.	2023	Saudi Arabia	Cross-sectional	476	5–16	Atopic Dermatitis	QoL impairment; emotional distress; sleep disturbance	CDLQI
02	Andrade et al.	2020	Brazil	Cross-sectional	123	Parents	Childhood Vitiligo	Emotional distress in parents; psychosocial burden	QLCCDQ, FDLQI
03	Baloch et al.	2025	Pakistan	Cross-sectional	474	10–16	Atopic Dermatitis	Moderate–severe QoL impairment; psychosocial impact	CDLQI
04	Campos et al.	2017	Brazil	Cross-sectional	51	5–16	Atopic Dermatitis	QoL impairment; family impact	CDLQI, DFI
05	Campos-Muñoz et al.	2023	Spain	Cross-sectional	191	4–16	Pediatric skin diseases (AD, acne, warts)	QoL impairment; sleep disturbance; emotional distress	CDLQI
06	Chong et al.	2024	Singapore	Cross-sectional	25	Pediatric	Chronic skin diseases	HRQoL impairment; emotional distress	CDLQI
07	Cipolletta et al.	2018	Italy	Cross-sectional comparative	120 (60 NF1 + 60 controls)	6–17	Neurofibromatosis Type 1	Anxiety; social difficulties; reduced QoL	PedsQL 4.0
08	Costa et al.	2020	Brazil	Qualitative phenomenological	6 adolescents	12–18	Chronic skin diseases	Stigma; self-esteem; social isolation	Qualitative interviews
09	Day et al.	2025	United Kingdom	Qualitative (IPA)	8 dyads	10–16	Psoriasis	Stigma; adolescent distress; family impact	CDLQI, FDLQI
10	De Maeseneer et al.	2019	Belgium	Cross-sectional	50	<18	Severe chronic skin diseases	QoL; adaptation; parental experience	My Positive Health; Pelentsov
11	Fishbein et al.	2021	USA	Cross-sectional	180	5–17	Atopic dermatitis	Sleep disturbance; depression; anxiety	PROMIS; CDLQI
12	Fitch	2024	USA	Cross-sectional	1,671	8–17	Chronic pediatric skin disorders	Stigma; bullying; anxiety; depression	Skindex-Teen; PROMIS
13	Franz et al.	2025	Europe	Cross-sectional	417	10–17	Alopecia areata	Bullying; emotional distress	PedsQL; SDQ
14	Abeni et al.	2021	Italy	Multicenter cross-sectional	78 (48 < 18)	Pediatric + adult	Congenital ichthyosis	Family burden; caregiver QoL	FDLQI; FBI; (C)DLQI
15	Gunduz et al.	2017	Turkey	Case-control	120	NR	Atopic dermatitis (maternal impact)	OCD symptoms; maternal QoL	SF-36
16	Heapy et al.	2021	UK	Cross-sectional	180	∼10	Psoriasis & eczema	Parental stress; depression	CDLQI; FDLQI
17	Heapy et al.	2022	UK	Experimental (single-group)	7	4–12	Psoriasis & eczema	Parenting stress; psychological distress	CDLQI; FDLQI
18	Hemrajani et al.	2022	India	Cross-sectional	60	<16	Congenital ichthyosis	Poor QoL; family impact	CDLQI; DFI
19	Hughes et al.	2023	UK	Qualitative	23	8–11	Chronic skin conditions	Caregiver burden; mood; sleep problems	CDLQI; FDLQI
20	Kauppi et al.	2021	Finland	Nationwide cohort	70,584 + controls	<18	Atopic dermatitis	Eating disorders risk	Registry (ICD codes)
21	Kelly et al.	2021	UK	Cross-sectional	Pediatric sample	5–16	Atopic dermatitis	Emotional impact; sleep disturbance	SMFQ, SDQ
22	Kern et Al.	2021	United Kingdom	Cohort study	11,181	6 mons—18	Atopic dermatitis	Depression, anxiety, severe AD	SMFQ, SDQ
23	Khattak et al.	2025	Pakistan	Cross-sectional	Pediatric sample	≤18	Vitiligo	Anxiety; stigma	CDLQI
24	Kılıç & Kılıç	2023	Turkey	Cross-sectional	Pediatric sample	6–16	Psoriasis	Depression; stigma	CDLQI
25	Lai et al.	2024	USA	Psychometric validation	860	8–17	Chronic skin disorders	Stigma; anxiety; depression	PROMIS Pediatric Stigma
26	Lam et al.	2024	Hong Kong	Cross-sectional	Pediatric sample	6–17	Atopic dermatitis	Sleep disturbance; QoL impairment	CDLQI
27	Leong et al.	2022	Singapore	Cross-sectional	Pediatric sample	≤16	Atopic dermatitis	Emotional distress; family burden	CDLQI; IDQoL
28	Manzoni et al.	2012	Brazil	Cross-sectional	118	5–16	AD, vitiligo, psoriasis	QoL impairment	CDLQI
29	Manzoni et al.	2013	Brazil	Cross-sectional	118	5–16	AD, vitiligo, psoriasis	Caregiver anxiety/depression	CDLQI; BDI; HAS
30	Min et al.	2023	South Korea	Cross-sectional	Pediatric sample	≤18	Atopic dermatitis	Psychological distress; sleep issues	CDLQI
31	Miniksar et al.	2023	Turkey (tertiary clinic)	Cross-sectional	82	6–17	Psoriasis	Anxiety, depression, stigma	CDLQI
32	Mohamed & Aboelmagd	2022	Egypt (dermatology clinic)	Cross-sectional	100	6–16	Atopic dermatitis	Emotional distress, sleep disturbance	CDLQI
33	Paller et al.	2024	USA/Canada (32 centers)	Multicenter cross-sectional	1,671	8–17	Chronic skin disorders	Stigma, anxiety, depression	Skindex-Teen; PROMIS
34	Salman et al.	2018	Saudi Arabia (hospital-based)	Cross-sectional	94	5–16	Vitiligo	Self-esteem, emotional impact	CDLQI
35	Seivright et al.	2021	UK (dermatology clinics)	Cross-sectional	130	8–16	Atopic dermatitis	Anxiety, sleep problems	CDLQI; SDQ
36	Soon et al.	2024	UK (specialist service)	Cross-sectional	122	8–16	Chronic dermatologic disorders	Attachment style, emotional/behavioral difficulties	SDQ
37	Sultan et al.	2025	Middle East (multicenter)	Cross-sectional	160	6–18	Psoriasis	Stigma, depressive symptoms	CDLQI
38	Sur et al.	2020	Romania (pediatric clinic)	Cross-sectional	64	0–16	Atopic dermatitis	Pruritus, sleep, mood	IDQoL; CDLQI
39	Xie & Liang	2022	Hong Kong	Qualitative	17	8–12	Atopic dermatitis	Self-stigma, emotional distress	SCORAD
40	Xu et al.	2019	Singapore	Multicenter cross-sectional	559	0–16	Atopic dermatitis	Emotional distress, caregiver burden	IDQoL; CDLQI; RAND-36
41	Żychowska et al.	2020	Poland	Cross-sectional	65	5–17	Psoriasis	Caregiver distress, social impact	FDLQI; CDLQI

Summary of study design, setting, population characteristics, and key methodological features of the included studies.

Most quantitative studies used validated assessment tools including the Children's Dermatology Life Quality Index (CDLQI), Pediatric Quality of Life Inventory (PedsQL), Family Dermatology Life Quality Index (FDLQI), Hospital Anxiety and Depression Scale (HADS), and Children's Depression Inventory (CDI), while most qualitative studies used structured or semi-structured interviews.

### 3.3 Psychological outcomes

**Figure 2:**
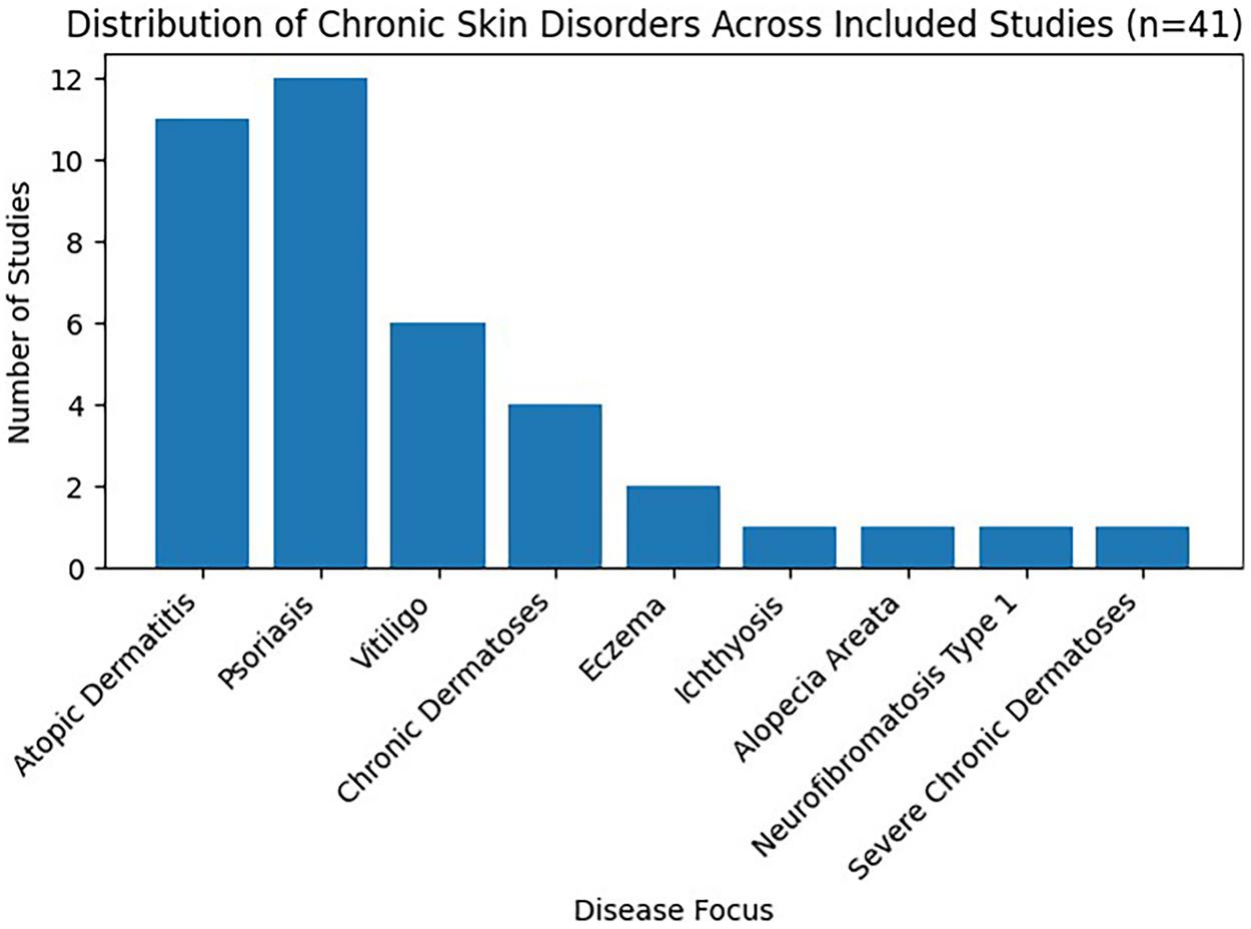
Distribution chronic skin disorder across included studies (*n* = 41). Figure 2 illustrates the distribution of chronic skin disorders investigated across the 41 included studies. Psoriasis (*n* = 12) and atopic dermatitis (*n* = 11) were the most frequently studied conditions, followed by vitiligo (*n* = 6), chronic dermatoses (*n* = 4), and eczema (*n* = 2). Ichthyosis, alopecia areata, neurofibromatosis type 1, and severe chronic dermatoses were each represented by one study.

Psychological distress was consistently associated with chronic skin disorders across the included studies (see Table 2 and Figure 2). Several studies identified elevated levels of anxiety, depression, stigma, and emotional burden among pediatric patients with chronic skin disorders, though the predominantly cross-sectional designs preclude causal interpretation (19, 22, 30). Higher anxiety and depressive symptoms were frequently associated with greater disease severity in atopic dermatitis (19). Stigma and social visibility were identified as major determinants of psychological distress in children with chronic dermatologic conditions (30, 47). Longitudinal evidence further demonstrated an increased risk of depressive and internalizing symptoms among children with severe atopic dermatitis (1).

**Table 2:** Psychological outcomes reported in included studies (*n* = 41).

No.	Author (Year)	Disease focus	Main psychological outcomes	Measurement instruments	Key psychological findings
01	Aldosari et al. (19)	Atopic dermatitis	Anxiety, depression, stress	DASS-21	Higher disease severity associated with elevated anxiety and depression
02	Andrade et al. (20)	Vitiligo	Parental emotional distress	QLCCDQ, FDLQI	Greater BSA linked to worse caregiver emotional QoL
03	Baloch et al. (58)	Psoriasis	Stigma, low self-esteem	CDLQI	Visible lesions associated with higher stigma scores
04	Campos et al. (21)	Atopic dermatitis	Anxiety, behavioral issues	SDQ	Increased emotional and conduct problems
05	Campos-Muñoz et al. (22)	Chronic dermatoses	Depression, social withdrawal	CDI	Moderate depressive symptoms reported
06	Chong et al. (23)	Eczema	Anxiety, sleep disturbance	PedsQL	Poor sleep correlated with anxiety levels
07	Cipolletta et al. (24)	Neurofibromatosis type 1	Social anxiety	SAFA, CBCL	Higher anxiety vs. controls
08	Costa et al. (25)	Psoriasis	Stress, peer difficulties	SDQ	Peer problems significantly elevated
09	Day et al. (26)	Psoriasis	Stigma, shame, parental stress	CDLQI, FDLQI	Visibility more distressing than symptoms
10	De Maeseneer et al. (27)	Severe chronic dermatoses	Resilience, parental burden	Modified Positive Health Questionnaire	Parents reported high psychosocial needs
11	Fishbein et al. (28)	Atopic dermatitis	Depression, anxiety	PHQ-9, GAD-7	Psychological distress correlated with itch severity
12	Fitch (29)	Alopecia areata	Self-image issues	CDLQI	Hair loss strongly linked to self-esteem reduction
13	Franz et al. (30)	Psoriasis	Anxiety, depressive symptoms	HADS	Moderate anxiety levels observed
14	Abeni et al. (55)	Ichthyosis	Emotional distress	FDLQI	Severe cases linked to higher family stress
15	Gunduz et al. (31)	Vitiligo	Depression, social anxiety	CDI	Emotional impact significant in adolescents
16	Heapy et al. (32)	Psoriasis	Shame, stigma	CDLQI	Stigma predicted poorer mental health
17	Heapy et al. (33)	Psoriasis	Treatment-related stress	IPA interviews	Emotional tension during adolescence
18	Hemrajani et al. (34)	Atopic dermatitis	Behavioral problems	SDQ	Elevated hyperactivity and emotional symptoms
19	Hughes et al. (35)	Psoriasis	Self-esteem reduction	CDLQI	Greater lesion visibility increased distress
20	Kauppi et al. (36)	Chronic dermatoses	Anxiety	PedsQL	HRQoL impairment correlated with anxiety
21	Kelly et al. (37)	Atopic dermatitis	Emotional burden	CDLQI	Moderate psychosocial impairment
22	Kern et al. (1)	Atopic Dermatitis	Depression; internalizing behaviors	SMFQ, SDQ	Higher risk of depression and internalizing symptoms
23	Khattak et al. (38)	Psoriasis	Depression	PHQ-9	Higher depression in severe cases
24	Kılıç & Kılıç (39)	Vitiligo	Stigma, social withdrawal	CDLQI	Visibility predicted social isolation
25	Lai et al. (40)	Chronic skin disease	Stigma, anxiety	PROMIS	Stigma strongly correlated with depression
26	Lam et al. (41)	Eczema	Peer bullying	SDQ	Bullying increased emotional distress
27	Leong et al. (42)	Atopic dermatitis	Sleep disturbance, anxiety	PedsQL	Sleep loss worsened emotional function
28	Manzoni et al. (43)	Atopic dermatitis	Depression symptoms	CDLQI	Higher impairment in AD vs. vitiligo
29	Manzoni et al. (44)	Psoriasis	Social embarrassment	CDLQI	Lesion visibility linked to stigma
30	Min et al. (45)	Atopic dermatitis	Anxiety	GAD-7	Anxiety correlated with disease activity
31	Miniksar et al. (46)	Vitiligo	Emotional distress	CDLQI	QoL impairment significant
32	Alkady & Aboelmgd (56)	Psoriasis	Stress, depression	DASS-21	Moderate psychological burden
33	Paller et al. (47)	Atopic dermatitis	Stigma	PROMIS Stigma	Stigma major determinant of mental health
34	Salman et al. (48)	Vitiligo	Social anxiety	CDLQI	Emotional impact significant
35	Seivright et al. (49)	Psoriasis	Stigma	DLQI	Stigma associated with social avoidance
36	Soon et al. (50)	Chronic dermatoses	Attachment insecurity	CAI, SDQ	Insecure attachment linked to distress
37	Sultan et al. (12)	Psoriasis	Emotional burden	CDLQI	Higher severity worsened psychological impact
38	Sur et al. (51)	Atopic dermatitis	Sleep disturbance	PedsQL	Sleep impairment linked to anxiety
39	Xie & Liang (52)	Atopic dermatitis	Self-stigma	Qualitative interviews	Stigma worsened psychosocial wellbeing
40	Xu et al. (53)	Psoriasis	Anxiety	HADS	Significant emotional impairment
41	Żychowska et al. (54)	Vitiligo	Depression, stigma	CDLQI	Social stigma increased depressive symptoms

Overview of psychological outcomes, including emotional distress, anxiety, depression, stigma, and behavioral difficulties, among pediatric patients with chronic skin disorders.

Sleep disturbance and emotional instability were common, particularly among those with severe pruritus. In psoriasis, depressive symptoms independent of disease severity were greater. Emotional vulnerability was greater in females and adolescents (22).

Most studies found impaired HRQoL among pediatric patients with chronic skin disorders (see Table 3). For example, HRQoL scores were significantly impaired in children with atopic dermatitis, especially in sleep and symptom domains (34). Moderate-to-severe emotional impairment and reduced peer functioning among children with chronic dermatoses was also found (22).

**Table 3:** Health-Related quality of life (HRQoL) outcomes in included studies (*n* = 41).

No.	Author (Year)	Disease focus	HRQoL domains affected	Assessment instruments	Key HRQoL findings
01	Aldosari et al. (19)	Atopic dermatitis	Emotional, sleep, symptoms	CDLQI	Severe AD significantly worsened emotional and sleep domains
02	Andrade et al. (20)	Vitiligo	Emotional, social, family burden	QLCCDQ, FDLQI	Larger BSA associated with poorer caregiver emotional QoL
03	Baloch et al. (58)	Psoriasis	Emotional, social functioning	CDLQI	Visible psoriasis linked to reduced social QoL
04	Campos et al. (21)	Atopic dermatitis	Symptoms, feelings, sleep	CDLQI	Higher SCORAD correlated with worse QoL
05	Campos-Muñoz et al. (22)	Chronic dermatoses	Emotional, peer relations	PedsQL	Moderate-to-severe impairment in emotional domains
06	Chong et al. (23)	Eczema	Physical, emotional, school	PedsQL	Sleep disturbance reduced school functioning
07	Cipolletta et al. (24)	NF1	Physical, social, school	PedsQL	NF1 patients had poorer overall QoL vs. controls
08	Costa et al. (25)	Psoriasis	Emotional, social participation	CDLQI	Social functioning strongly impaired
09	Day et al. (26)	Psoriasis	Emotional, social, family	CDLQI, FDLQI	Visibility had greater QoL impact than symptoms
10	De Maeseneer et al. (27)	Severe dermatoses	Physical, mental, daily functioning	Modified My Positive Health	Despite severity, some children reported adaptive QoL
11	Fishbein et al. (28)	Atopic dermatitis	Emotional, sleep	CDLQI	Itch severity associated with worse QoL
12	Fitch (29)	Alopecia areata	Self-image, social	CDLQI	Hair loss significantly impaired self-image domain
13	Franz et al. (30)	Psoriasis	Emotional, leisure	DLQI	Moderate HRQoL impairment
14	Abeni et al. (55)	Ichthyosis	Emotional, family burden	FDLQI	Severe disease increased family QoL burden
15	Gunduz et al. (31)	Vitiligo	Emotional, social	CDLQI	Adolescents reported social withdrawal
16	Heapy et al. (32)	Psoriasis	Emotional, self-esteem	CDLQI	Stigma strongly reduced QoL
17	Heapy et al. (33)	Psoriasis	Emotional, daily routines	CDLQI	Adolescents struggled with autonomy and treatment
18	Hemrajani et al. (34)	Atopic dermatitis	Symptoms, sleep	CDLQI	Sleep disruption worsened QoL
19	Hughes et al. (35)	Psoriasis	Emotional, peer interaction	CDLQI	Visible lesions reduced peer-related QoL
20	Kauppi et al. (36)	Chronic dermatoses	Physical, emotional	PedsQL	Moderate QoL reduction observed
21	Kelly et al. (37)	Atopic dermatitis	Emotional, school	CDLQI	QoL impairment correlated with severity
22	Kern et al. (1)	Atopic Dermatitis	NR	SMFQ, SDQ	NR
23	Khattak et al. (38)	Psoriasis	Emotional, social	CDLQI	Severe cases had worse HRQoL
24	Kılıç & Kılıç (39)	Vitiligo	Emotional, social	CDLQI	Visibility significantly affected QoL
25	Lai et al. (40)	Chronic skin disease	Emotional, peer, mental health	PROMIS	Stigma predicted poorer HRQoL
26	Lam et al. (41)	Eczema	Emotional, school	PedsQL	Bullying reduced school-related QoL
27	Leong et al. (42)	Atopic dermatitis	Sleep, emotional	PedsQL	Sleep impairment strongly reduced HRQoL
28	Manzoni et al. (43)	Atopic dermatitis	Symptoms, leisure, relationships	CDLQI	AD had greater QoL impairment vs. vitiligo
29	Manzoni et al. (44)	Psoriasis	Emotional, visibility impact	CDLQI	Visible lesions worsened QoL
30	Min et al. (45)	Atopic dermatitis	Emotional, symptoms	CDLQI	Disease activity associated with poorer QoL
31	Miniksar et al. (46)	Vitiligo	Emotional, social	CDLQI	Moderate HRQoL impairment
32	Alkady & Aboelmgd (56)	Psoriasis	Emotional, daily functioning	DLQI	QoL decreased with disease severity
33	Paller et al. (47)	Atopic dermatitis	Emotional, peer, sleep	PROMIS	Stigma and severity predicted poor HRQoL
34	Salman et al. (48)	Vitiligo	Emotional, social	CDLQI	Visible areas significantly impaired QoL
35	Seivright et al. (49)	Psoriasis	Emotional, social	DLQI	Stigma associated with poorer HRQoL
36	Soon et al. (50)	Chronic dermatoses	Emotional, peer functioning	SDQ	Insecure attachment linked to reduced QoL
37	Sultan et al. (12)	Psoriasis	Emotional, school	CDLQI	Severity predicted poorer school functioning
38	Sur et al. (51)	Atopic dermatitis	Sleep, emotional	PedsQL	Sleep disturbance worsened QoL
39	Xie & Liang (52)	Atopic dermatitis	Emotional, social identity	Qualitative interviews	Self-stigma deeply impaired psychosocial QoL
40	Xu et al. (53)	Atopic dermatitis	Symptoms, feelings	CDLQI	Greater severity associated with poorer QoL
41	Żychowska et al. (54)	Psoriasis	Emotional, caregiver burden	FDLQI	Childhood psoriasis had major caregiver QoL impact

Distribution of affected HRQoL domains, key findings, and validated instruments used to assess quality of life in pediatric dermatology populations.

Similarly, strong associations between disease severity and worse CDLQI scores in atopic dermatitis were noted (19, 53). In pediatric psoriasis, significant emotional and social QoL impairment related to lesion visibility and disease severity were found (12, 44). Across conditions, visible lesions, symptom burden, sleep disturbance, stigma were associated with decreased HRQoL.

### 3.4 Family and caregiver impact

Family and caregiver burden was frequently reported across the included studies (see Table 4). Correlations between child and caregiver quality of life scores for dermatologic conditions were shown (34). Caregivers of children with vitiligo and ichthyosis experienced emotional and financial strain (20, 55). Substantial parental stress and sleep disruption among families of children with atopic dermatitis were reported (19, 42). Across conditions, caregivers experienced emotional distress, reduced quality of life, financial strain, and psychosocial stress.

**Table 4:** Family impact and caregiver burden in included studies (*n* = 41).

No.	Author (Year)	Disease focus	Family impact domains	Assessment instruments
01	Aldosari et al. (19)	Atopic dermatitis	Parental stress; sleep disruption; emotional burden	CDLQI
02	Andrade et al. (20)	Vitiligo	Emotional distress; caregiver QoL impairment	FDLQI; QLCCDQ
03	Baloch et al. (58)	Chronic dermatoses	Caregiver burden; psychological stress	Not specified
04	Campos et al. (21)	Atopic dermatitis	Family emotional burden; sleep disturbance	DFI; CDLQI
05	Campos-Muñoz et al. (22)	Atopic dermatitis	Caregiver emotional stress; QoL impairment	FDLQI
06	Chong et al. (23)	Atopic dermatitis	Caregiver stress; sleep loss	FDLQI
07	Cipolletta et al. (24)	Neurofibromatosis type 1	Parental psychosocial stress	PedsQL
08	Costa et al. (25)	Chronic skin diseases	Parental emotional adaptation	Qualitative interviews
09	Day et al. (26)	Psoriasis	Parental anxiety; guilt; family tension	FDLQI; CDLQI
10	De Maeseneer et al. (27)	Severe chronic skin disorders	Unmet psychosocial needs; family strain	Pelentsov Scale
11	Fishbein et al. (28)	Atopic dermatitis	Caregiver QoL impairment	FDLQI
12	Fitch (29)	Dermatologic conditions	Family stress	Not specified
13	Franz et al. (30)	Chronic dermatoses	Family coping challenges	Not specified
14	Abeni et al. (55)	Ichthyosis	Financial burden; time burden; emotional distress	FDLQI; FBI
15	Gunduz et al. (31)	Psoriasis	Caregiver emotional distress	FDLQI
16	Heapy et al. (32)	Alopecia areata	Parental distress	FDLQI
17	Heapy et al. (33)	Alopecia areata	Family coping; caregiver burden	FDLQI
18	Hemrajani et al. (34)	Dermatologic conditions	Parental QoL burden	FDLQI
19	Hughes et al. (35)	Psoriasis	Family psychosocial strain	FDLQI
20	Kauppi et al. (36)	Atopic dermatitis	Family QoL reduction	FDLQI
21	Kelly et al. (37)	Psoriasis	Caregiver stress	FDLQI
22	Kern (1)	Atopic dermatitis	Family stress	FDLQI
23	Khattak et al. (38)	Atopic dermatitis	Caregiver burden	FDLQI
24	Kılıç & Kılıç (39)	Atopic dermatitis	Parental anxiety	FDLQI
25	Lai et al. (40)	Skin diseases	Family stigma; social strain	PROMIS
26	Lam et al. (41)	Atopic dermatitis	Family psychological stress	FDLQI
27	Leong et al. (42)	Atopic dermatitis	Caregiver mental health decline	FDLQI
28	Manzoni et al. (43)	AD, psoriasis, vitiligo	Caregiver anxiety/depression	CDLQI
29	Manzoni et al. (44)	AD, psoriasis, vitiligo	Caregiver psychological distress	BDI; HAS
30	Min et al. (45)	Dermatologic disorders	Parental QoL burden	FDLQI
31	Miniksar et al. (46)	Atopic dermatitis	Family QoL impact	FDLQI
32	Alkady & Aboelmgd (56)	Atopic dermatitis	Caregiver stress; emotional strain	FDLQI
33	Paller et al. (47)	Atopic dermatitis	Caregiver QoL impairment	FDLQI
34	Salman et al. (48)	Chronic dermatoses	Caregiver QoL reduction	FDLQI
35	Seivright et al. (49)	Psoriasis	Family stigma; caregiver distress	FDLQI
36	Soon et al. (50)	Chronic dermatologic disorders	Family coping and resilience	CAI; SDQ
37	Sultan et al. (12)	Chronic dermatoses	Parental psychological impact	Not specified
38	Sur et al. (51)	Atopic dermatitis	Family emotional burden	IDQoL; CDLQI
39	Xie & Liang (52)	Atopic dermatitis	Family conflict; stigma	SCORAD
40	Xu et al. (53)	Atopic dermatitis	Caregiver mental and physical health decline	RAND-36; IDQoL
41	Żychowska et al. (54)	Psoriasis	Emotional distress; financial strain	FDLQI

Summary of reported psychosocial, emotional, and functional effects of pediatric skin diseases on parents, caregivers, and family systems.

### 3.5 Risk and protective factors

#### 3.5.1 Risk factors

Several studies identified clinical and psychosocial factors associated with poorer psychological outcomes (see Table 5). Across conditions, greater disease severity and symptom burden were consistently associated with worse mental health outcomes (12, 19, 22). Persistent pruritus and sleep disturbance were frequently linked to emotional distress in atopic dermatitis (51, 56). Visible lesions and social stigma were identified as significant risk factors in psoriasis and vitiligo (26, 44). Additional contributors included female sex, larger body surface area involvement, and disease chronicity.

**Table 5:** Risk and protective factors reported in included studies (*n* = 41).

No.	Author (Year)	Condition	Risk factors	Protective factors
01	Aldosari et al. (19)	Atopic dermatitis	Night itching; emotional distress; treatment burden	Access to tertiary care
02	Andrade et al. (20)	Vitiligo	Larger BSA; visible lesions; younger age	Older age; lower severity
03	Baloch et al. (58)	Chronic dermatoses	Disease severity; stigma	Family support
04	Campos et al. (21)	Atopic dermatitis	Higher SCORAD; pruritus; low income	Not specified
05	Campos-Muñoz et al. (22)	Atopic dermatitis	Severe AD; sleep disturbance	Treatment adherence
06	Chong et al. (23)	Atopic dermatitis	Moderate–severe AD; sleep loss	Social support
07	Cipolletta et al. (24)	NF1	Cognitive problems; visible neurofibromas	Family involvement
08	Costa et al. (25)	Chronic skin diseases	Social stigma	Peer acceptance
09	Day et al. (26)	Psoriasis	Visible lesions; bullying; parental anxiety	Family communication; coping
10	De Maeseneer et al. (27)	Severe dermatoses	Chronicity; high treatment burden	Multidisciplinary care
11	Fishbein et al. (28)	Atopic dermatitis	Severe AD; poor sleep	Integrated care
12	Fitch (29)	Dermatologic disorders	Emotional vulnerability	Not specified
13	Franz et al. (30)	Chronic dermatoses	Disease visibility	Resilience
14	Abeni et al. (55)	Ichthyosis	Severe disease; recurrent infections	Milder severity
15	Gunduz et al. (31)	Psoriasis	Disease severity; social stigma	Family support
16	Heapy et al. (32)	Alopecia areata	Hair loss visibility; peer reactions	Coping strategies
17	Heapy et al. (33)	Alopecia areata	Stigma; loss of control	Psychological support
18	Hemrajani et al. (34)	Dermatologic disorders	Symptom burden	Social support
19	Hughes et al. (35)	Psoriasis	Stigma; treatment uncertainty	Acceptance coping
20	Kauppi et al. (36)	Atopic dermatitis	Severe eczema; itching	Disease management
21	Kelly et al. (37)	Psoriasis	Visible lesions	Peer support
22	Kern (1)	Atopic dermatitis	Persistent AD activity	Family support
23	Khattak et al. (38)	Atopic dermatitis	Severe AD; sleep disturbance	Parental education
24	Kılıç & Kılıç (39)	Atopic dermatitis	Parental anxiety; chronic symptoms	Family cohesion
25	Lai et al. (40)	Skin diseases	High stigma; female sex; severity	Peer discussion
26	Lam et al. (41)	Atopic dermatitis	Severe AD; long duration	Social support
27	Leong et al. (42)	Atopic dermatitis	Severe AD; sleep loss	Psychological care
28	Manzoni et al. (43)	AD/psoriasis/vitiligo	Large affected BSA; visible lesions	Vitiligo diagnosis
29	Manzoni et al. (44)	AD/psoriasis/vitiligo	Severe disease; visible areas	Not specified
30	Min et al. (45)	Dermatologic disorders	Disease severity; stigma	Support networks
31	Miniksar et al. (46)	Atopic dermatitis	Severe eczema; pruritus	Treatment control
32	Alkady & Aboelmgd (56)	Atopic dermatitis	Chronic itching; sleep disturbance	Education programs
33	Paller et al. (47)	Atopic dermatitis	Severe AD; symptom burden	Early intervention
34	Salman et al. (48)	Chronic dermatoses	Disease chronicity	Family resilience
35	Seivright et al. (49)	Psoriasis	Stigma; adolescent transition	Psychological therapy
36	Soon et al. (50)	Chronic dermatoses	Insecure attachment	Secure attachment
37	Sultan et al. (12)	Chronic dermatoses	Disease severity	Social support
38	Sur et al. (51)	Atopic dermatitis	High SCORAD; pruritus	Mild disease
39	Xie & Liang (52)	Atopic dermatitis	Self-stigma; bullying; visible rash	Family/peer support
40	Xu et al. (53)	Atopic dermatitis	Moderate–severe AD; itching	Coping strategies
41	Żychowska et al. (54)	Psoriasis	Female sex; household burden	Not specified

Identified clinical, psychosocial, and environmental factors associated with increased vulnerability or resilience among affected children and their families.

#### 3.5.2 Protective factors

Protective factors included support from family and peers, effective control of the disease, coping, and access to integrated dermatologic and psychological care (27, 31, 57). Psychological support services and coping were linked to emotional resilience in a few studies.

**Figure 3:**
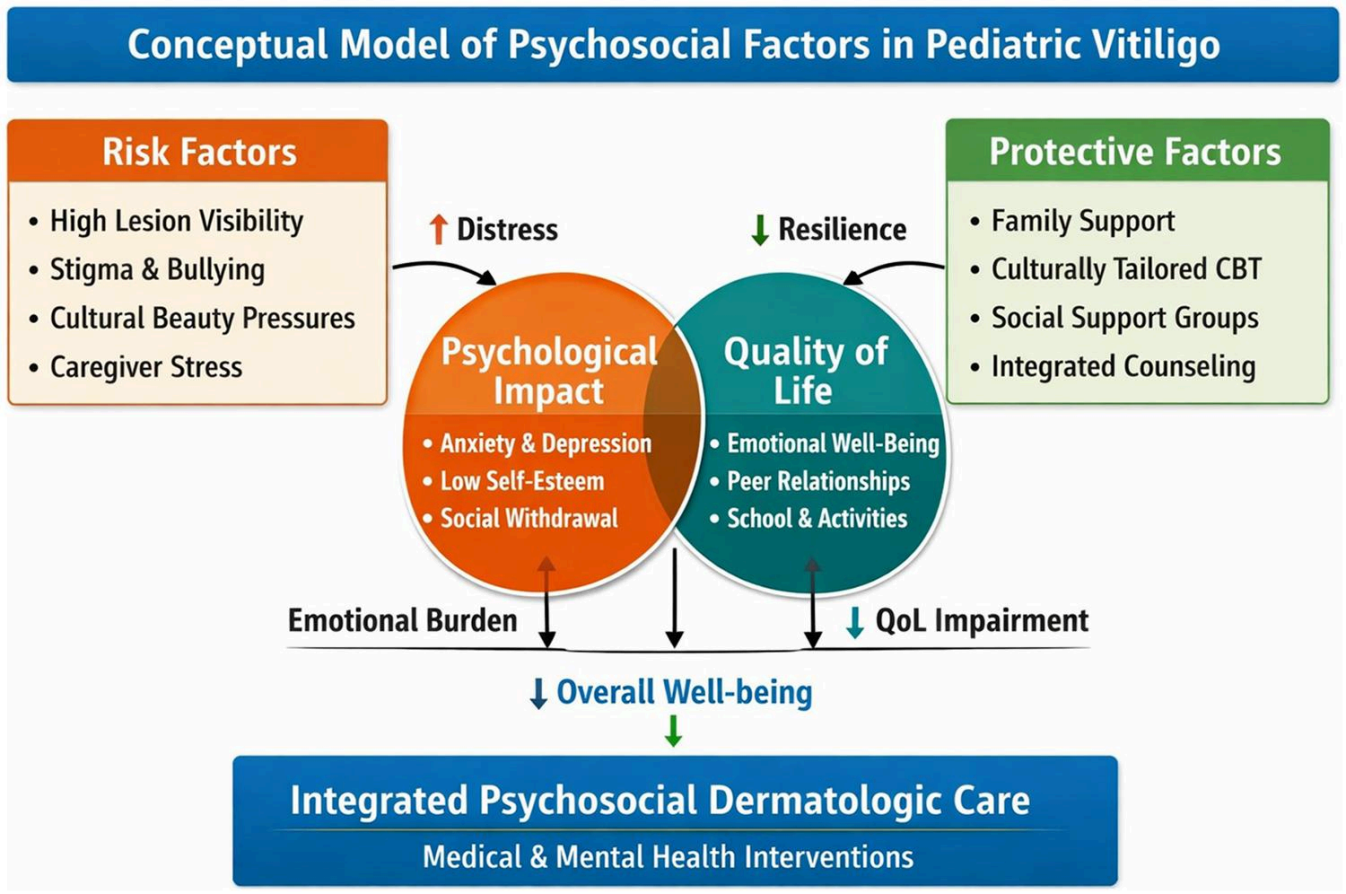
Conceptual framework of risk and protective factors influencing psychological outcomes and HRQoL. This conceptual model illustrates the interactions between disease-related risk factors, protective psychosocial resources, psychological distress, and health-related quality of life in pediatric patients with chronic skin disorders, highlighting the role of integrated dermatologic and mental health care in improving overall well-being.

Overall, “the studies suggest that severity, burden, disability, and social stigma of disease were associated with vulnerability to psychological distress, while family support, coping, and integrated care may mitigate adverse effects” (Figure 3).

### 3.6 Risk of bias summary and methodological quality

A total of 41 studies were included in the methodological appraisal. Overall methodological quality ranged from moderate to high. Quality was assessed using JBI checklists for quantitative studies and CASP for qualitative studies. Twelve studies were classified as high quality, while the majority were rated as moderate or moderate–high quality. No study was judged to be critically low-quality requiring exclusion from synthesis (see Table 6).

**Table 6:** Quality appraisal of included studies (*n* = 41).

No.	Author (Year)	Study design	Appraisal tool used	Key quality strengths	Main limitations	Overall quality
01	Aldosari et al. (19)	Multicenter cross-sectional	JBI	Large national sample; validated Arabic CDLQI	No objective severity correlation	High
02	Andrade et al. (20)	Prospective cross-sectional	JBI	Validated QLCCDQ & FDLQI; IRB approval	Parent-reported severity; cross-sectional	Moderate–High
03	Baloch et al. (58)	Cross-sectional	JBI	Validated tools; appropriate statistics	Single-center; self-report bias	Moderate
04	Campos et al. (21)	Cross-sectional	JBI	SCORAD & CDLQI used; statistical correlations	Small sample; single center	Moderate
05	Campos-Muñoz et al. (22)	Cross-sectional	JBI	Validated QoL measures	Cross-sectional design	Moderate
06	Chong et al. (23)	Cross-sectional	JBI	Validated psychological tools	Cross-sectional; self-report	Moderate–High
07	Cipolletta et al. (24)	Comparative cross-sectional	JBI	Control group; validated scales	Limited longitudinal data	High
08	Costa et al. (25)	Qualitative phenomenological	CASP	Clear analytic framework; ethical approval	Small sample	High
09	Day et al. (26)	Qualitative IPA	CASP/JARS-Qual	Reflexivity; audit trail; multi-reviewer coding	Small dyad sample	High
10	De Maeseneer et al. (27)	Multicenter mixed-methods	JBI	Multidisciplinary design; validated tools	Small sample size	Moderate–High
11	Fishbein et al. (28)	Cross-sectional	JBI	Validated measures; statistical rigor	Single-center	Moderate
12	Fitch (29)	Cross-sectional	JBI	Standardized psychological tools	Limited methodological reporting	Moderate
13	Franz et al. (30)	Cross-sectional	JBI	Large sample; validated scales	Cross-sectional	Moderate–High
14	Abeni et al. (55)	Multicenter cross-sectional	JBI	Disease severity scoring; validated FDLQI	No longitudinal follow-up	High
15	Gunduz et al. (31)	Cross-sectional	JBI	Validated QoL instruments	Single-center	Moderate
16	Heapy et al. (32)	Cross-sectional	JBI	Standardized tools	Self-reported data	Moderate
17	Heapy et al. (33)	Cross-sectional	JBI	Validated psychological scales	Cross-sectional	Moderate
18	Hemrajani et al. (34)	Cross-sectional	JBI	Appropriate statistics	Small sample	Moderate
19	Hughes et al. (35)	Qualitative	CASP	Clear methodology; ethical approval	Small sample	High
20	Kauppi et al. (36)	Cross-sectional	JBI	Validated eczema scales	Single-center	Moderate
21	Kelly et al. (37)	Cross-sectional	JBI	Validated QoL tools	No control group	Moderate
22	Kern (1)	Cohort study	JBI	Validated mental health tools	Limited ethnic diversity	High quality
23	Khattak et al. (38)	Cross-sectional	JBI	Standardized tools	Cross-sectional	Moderate
24	Kılıç & Kılıç (39)	Cross-sectional	JBI	Validated anxiety scales	Single-center	Moderate
25	Lai et al. (40)	Psychometric validation	JBI	IRT modeling; CFA; large sample	Cross-sectional	High
26	Lam et al. (41)	Cross-sectional	JBI	Validated measures	Limited adjustment for confounders	Moderate
27	Leong et al. (42)	Cross-sectional	JBI	Large pediatric sample	Cross-sectional	Moderate–High
28	Manzoni et al. (43)	Cross-sectional	JBI	CDLQI; regression analysis	Single-center	Moderate
29	Manzoni et al. (44)	Cross-sectional	JBI	Validated BDI & HAS	Cross-sectional	Moderate
30	Min et al. (45)	Cross-sectional	JBI	Large sample; validated tools	Self-report bias	Moderate–High
31	Miniksar et al. (46)	Cross-sectional	JBI	Validated eczema measures	Small sample	Moderate
32	Alkady & Aboelmgd (56)	Cross-sectional	JBI	Appropriate statistical analysis	Single-center	Moderate
33	Paller et al. (47)	Multicenter study	JBI	Large sample; standardized measures	Cross-sectional	High
34	Salman et al. (48)	Cross-sectional	JBI	Validated instruments	Cross-sectional	Moderate
35	Seivright et al. (49)	Qualitative	CASP	Clear thematic framework	Small sample	High
36	Soon et al. (50)	Cross-sectional	JBI	Standardized attachment measures	Cross-sectional	Moderate–High
37	Sultan et al. (12)	Cross-sectional	JBI	Validated HRQoL tools	Limited confounder reporting	Moderate
38	Sur et al. (51)	Cross-sectional	JBI	SCORAD; validated QoL scales	Small sample	Moderate
39	Xie & Liang (52)	Qualitative	CASP/COREQ	Dual coding; thematic rigor	Secondary analysis	High
40	Xu et al. (53)	Multicenter cross-sectional	JBI	Large sample; validated instruments	Cross-sectional	High
41	Żychowska et al. (54)	Cross-sectional	JBI	Validated FDLQI; statistical rigor	Single-center	Moderate

Assessment of study quality, risk of bias, and methodological rigor based on standardized appraisal criteria.

Common across quantitative studies were restrictions such as cross-sectional investigations that made it difficult to make causal inferences, single-center recruitment, lack of controls, and use of self-reported psychological and HRQoL measures. Some papers did not report whether they adjusted for confounders, or make it clear in their methodology. Qualitative studies were particularly small, and limited in reflexivity, but most had clear analytic frameworks and ethical oversight.

Conversely, multicenter, in cohort studies, and psychometric studies were more methodologically robust, indicating they used larger samples, validated instruments, and more complex statistics such as item response theory modeling and confirmatory factor analysis.

Risk of bias was considered during synthesis. Greater interpretive emphasis was placed on findings from studies rated as high methodological quality; however, no formal weighting or meta-analytic adjustment was applied. Despite methodological heterogeneity, the consistency of findings across moderate- to high-quality studies strengthens confidence in the overall conclusions of this review.

## 4 Discussion

This systematic review analyzed the psychological impact and health-related quality of life (HRQoL) amongst children presenting with chronic skin disorders. A total of 41 studies published from 2010 to 2025 that report these variables were included. Overall, evidence suggests significant emotional distress, impact on social functioning, and diminished well-being across a range of conditions, (e.g., atopic dermatitis, psoriasis, vitiligo, alopecia areata, ichthyosis) (19, 22, 30, 44).

### 4.1 Psychological impact of chronic skin disorders

Psychological morbidity was consistently associated with cutaneous conditions across studies. Elevated anxiety, depressive symptoms, emotional distress, and lower self-esteem were frequently reported among affected children and adolescents, though causal directionality cannot be established given the cross-sectional nature of most included studies (19, 22, 30).

In longitudinal studies of severe atopic dermatitis, a nearly two-fold increased risk of depressive and internalizing symptoms across childhood and adolescence was reported (1). Adolescents appear to be at heightened risk, particularly in the presence of visible lesions where stigma and peer-related concerns contribute to emotional distress.

Adolescents tend to be at heightened risk particularly with more visible lesions where stigma and peer related concerns are driving emotional distress (30, 44).

Intriguingly, some studies suggest that psychological distress may prevail in the face of moderate clinical severity, psychosocial burden is not fully accounted by dermatological indicators (19, 22).

### 4.2 Health-related quality of life impairment

The majority of studies reported impaired HRQoL in young patients (12, 22, 34). Emotional functioning, school participation, peer relationships and sleep were most often affected.

In atopic dermatitis, symptom burden — particularly pruritus and sleep disturbance — was closely linked to reduced quality of life (19, 34). Similarly, studies in psoriasis in the pediatric population noted important impairment to emotional and social HRQoL associated with lesion visibility and disease severity (12, 44).

In alopecia areata and vitiligo, quality-of-life impairment was strongly associated with social visibility and stigma rather than physical discomfort alone (20, 30).

Therapeutic regimens ranging from topical agents and phototherapy to biologics requiring laboratory monitoring and invasive procedures impose considerable time, financial, and emotional demands on patients and caregivers. While the primary literature did not consistently isolate treatment burden as an independent outcome domain, several included studies documented treatment-related stress as a contributing factor to caregiver burden and reduced HRQoL. Future systematic reviews should explicitly incorporate treatment burden as a primary outcome domain to more comprehensively capture the full psychosocial impact of chronic skin disease management.

### 4.3 Family and caregiver burden

Family impact was consistently documented across multiple conditions. Strong correlations between child and caregiver HRQoL scores were reported (34). Caregiver emotional strain and compromised family well-being in vitiligo and ichthyosis were also described (20, 55).

In atopic dermatitis, parental stress and sleep disruption were noted, again reflecting the chronic, relapsing nature of the condition (19, 42).

These findings support family-centered care approaches in pediatric dermatology.

### 4.4 Risk and protective factors

Higher severity, chronicity of symptoms, and visible skin lesions were the most frequently identified risk factors associated with negative psychological outcomes (12, 19, 22). In some studies, stigma and peer challenges heightened emotional susceptibility (30, 44).

Conversely, protective factors included strong family support, effective disease control, coping strategies, and access to multidisciplinary care (27, 31, 57). Studies that highlighted integrated dermatologic and psychological approaches reported better emotional resilience and improved HRQoL results.

### 4.5 Methodological considerations

Most studies were cross-sectional in design and therefore limited in causal inference. Only a few employed longitudinal methods, including a population-based cohort study (1). Heterogeneity in outcome measures and limited reporting of confounder adjustment were additional common methodological challenges.

Results that were consistent across moderate- to high-quality studies increases confidence in the overall conclusions.

### 4.6 Implications for clinical practice

The implications of our findings for practice are substantial. Routine psychological screening may be appropriate for routine dermatological consultation with adolescents, as embedding screening in dermatology clinics may help improve the recognition and management of psychosocial distress, particularly for individuals with severe, visible and/or long-standing skin disease. Validated instruments such as the CDLQI, PedsQL and HADS may assist with this process, particularly if implemented in conjunction with screening interventions.

However, while widely validated, these instruments do not consistently capture treatment burden as an independent domain. In the current therapeutic landscape — characterized by targeted biologics and precision medicine — quantifying the psychosocial weight of active treatment vs. non-treatment or alternative sequencing is increasingly important. Future outcome measurement frameworks should therefore delineate disease burden from treatment burden to more accurately reflect the full patient experience.

As these psychosocial aspects appear to be so interrelated with the physical disease and vice versa, involvement of psychologists in a multidisciplinary care model alongside the dermatologists themselves, nurses and social workers/services will likely be essential to the provision of high-quality care for this population.

It must be acknowledged that a significant disparity exists between the ideal multidisciplinary care model described in this review and real-world healthcare infrastructure. Even within highly developed nations, access to dedicated psychologists, mental health allies, and specialized psychiatric services within dermatology settings remains limited. In lower- and middle-income countries, these resources are frequently absent entirely. Implementation of psychosocial care recommendations must therefore be adapted to the resource realities of each clinical context.

For clinicians operating within resource-limited settings, pragmatic alternatives to formal multidisciplinary care should be considered. These include leveraging primary care partnerships to facilitate routine psychosocial screening, utilizing validated digital health tools and app-based PROMs where specialist referral is unavailable, and engaging community-based support networks and patient advocacy groups as supplementary resources. Such locally adaptable approaches can extend the reach of psychosocial support beyond tertiary dermatology centers and offer actionable, internationally applicable guidance for clinicians operating under varied healthcare frameworks.

### 4.7 Research gaps and future directions

Despite growing awareness of psychosocial burden pertinent to pediatric dermatology, key deficits exist. The predominance of cross-sectional designs offers insufficient insights into causal sequelae and longitudinal psychological trajectories; additional longitudinal studies are needed to utilize such data to sketch developmental trajectories of distress.

Few studies rigorously determine long-term effectiveness of psychosocial or multidisciplinary programs, representing a critical gap for future work. Greater standardization of outcome measures would facilitate comparability across studies and future meta-analytic synthesis, and research from low and middle-income countries is under-represented.

### 4.8 Strengths and limitations of this review

#### 4.8.1 Strengths

Coverage of multiple databases, clear eligibility criteria, and use of structured methodological appraisal were strengths of this systematic review. The synthesis of recent evidence across a variety of dermatologic conditions allows for updated insights into psychosocial outcomes in the pediatric population.

#### 4.8.2 Limitations

Heterogeneity of study design, outcome measures, and reporting limited qualitative synthesis. Furthermore, the predominance of cross-sectional evidence hampers causal interpretation. Language restrictions and publication bias may have affected study selection.

### 4.9 Summary

This review highlights that among chronic cutaneous disorders in children, psychological distress, impaired HRQoL and burden on care givers are consistently reported as effects. These associations may be impacted by severity, symptom chronification, stigma and parent-child interaction. Integrated evaluation and treatment of dermatological and psychosocial concerns is warranted.

## 5 Conclusion

This systematic review synthesized 41 studies published between 2010 and 2025 on the psychological impact and HRQoL of pediatric patients with chronic skin disorders. Findings consistently indicate that chronic skin diseases are associated with significant emotional distress, impaired social functioning, and reduced overall well-being. The psychosocial burden frequently paralleled or exceeded physical symptom severity, with notable effects on children's development and daily functioning.

Conditions most frequently studied included atopic dermatitis, psoriasis, vitiligo, alopecia areata, hidradenitis suppurativa, and congenital ichthyosis. Disease severity, chronicity, pruritus, sleep disturbance, and lesion visibility were consistently associated with poorer psychological outcomes. Adolescents were particularly vulnerable, given the compounding effects of stigma, peer difficulties, and body image concerns.

Caregiver and family burden was also prominently documented. Parental stress and reduced quality of life were strongly correlated with child outcomes, underscoring the interdependence of pediatric chronic illness and family well-being, and supporting family-centered, multidisciplinary care models.

Most included studies were of moderate-to-high methodological quality; however, the predominance of cross-sectional designs and self-reported measures limits causal inference. The consistency of associations across diverse study populations and settings nonetheless strengthens confidence in the overall conclusions.

In summary, psychological well-being and HRQoL represent critical dimensions of burden in pediatric dermatology. Routine psychosocial assessment and integrated multidisciplinary support should be embedded in standard clinical care to improve long-term outcomes.

### 5.1 Recommendations

Using validated assessment tools like the CDLQI and PedsQL, routine mental health evaluation should be standard practice in pediatric dermatology, especially for patients at higher risk. There should be a coordinated system of care using a multi-disciplinary and family-centered approach to care for the linked physical and psychosocial needs of children with dermatological conditions. Particular attention should be paid to adolescents, and there should be targeted supports available to help adolescents with body image and peer-related issues. Providing assistance for eliminating the stigma attached to dermatological conditions will help improve emotional and quality of life outcomes for affected children, as will assisting in the optimal management of their dermatological disease.

### 5.2 Recommendations for future research

Future studies should prioritize longitudinal designs to clarify causal pathways and psychological trajectories. High-quality intervention trials evaluating psychosocial and family-based programs are needed. Standardization of outcome measures would improve comparability across studies. Additional research in underrepresented regions, as well as evaluation of digital mental health tools and integrated care models, may enhance global applicability and inform health system planning.

## Data Availability

The original contributions presented in the study are included in the article/Supplementary Material, further inquiries can be directed to the corresponding author.
